# Prognostic Value of Combined Aquaporin 3 and Aquaporin 5 Overexpression in Hepatocellular Carcinoma

**DOI:** 10.1155/2013/206525

**Published:** 2013-10-09

**Authors:** Xiaodong Guo, Ting Sun, Mei Yang, Zhiyan Li, Zhiwei Li, Yuejuan Gao

**Affiliations:** ^1^302 Hospital of PLA, Beijing 100039, China; ^2^Navy General Hospital, Beijing 100048, China

## Abstract

*Background*. Aquaporin (AQP) 3 and AQP 5 are involved in tumorigenesis and tumor progression of several tumor types. *Aim*. To investigate expression patterns and clinical significance of AQP3 and AQP5 in hepatocellular carcinoma (HCC). *Methods*. Immunohistochemistry was performed to detect the expression of AQP3 and AQP5 in HCC tissues. *Results*. Immunohistochemistry analysis showed the increased expression of AQP3 and AQP5 protein levels in HCC tissues compared with their adjacent nonneoplastic tissues (both *P* < 0.001). In addition, combined AQP3 and AQP5 protein expression was significantly associated with serum AFP (*P* = 0.008), tumor stage (*P* = 0.006), and tumor grade (*P* = 0.006). Moreover, HCC patients highly expressing both AQP3 and AQP5 proteins had worse 5-year disease-free survival and 5-year overall survival (*P* = 0.002 and 0.005, resp.). Furthermore, the Cox proportional hazards model showed that combined AQP3 and AQP5 protein expression was an independent poor prognostic factor for both 5-year disease-free survival (*P* = 0.009) and 5-year overall survival (*P* = 0.01) in HCC. *Conclusion*. Our data suggest for the first time that the aberrant expression of AQP3 and AQP5 proteins may be strongly related to tumor progression and prognosis in patients with HCC. The overexpression of AQP3 in combination with upregulation of AQP5 may be an unfavorable prognostic factor for HCC.

## 1. Introduction

Hepatocellular carcinoma (HCC) represents one of the most common tumors worldwide and ranks as the third cause of cancer-related death, especially in east Asia and sub-Saharan Africa [1]. In China, HCC accounts for about 110,000 deaths annually and is the second leading cause of cancer-related death among men [2]. In the USA and Europe, the incidence of HCC has also been increasing in the recent years. As a highly aggressive solid tumor, HCC is characterized by fast infiltrating growth, early metastasis, high-grade malignancy, and poor prognosis. Despite improvements in treatment modalities during the past few decades, the long-term survival remains unsatisfactory mainly because of a high incidence of postoperative metastasis and recurrence and the high resistance of HCC to chemotherapy [3]. Since tumor progression of HCC is a complicated process that is associated with cumulative genomic alterations, the abnormal expression of oncogenes and tumor suppressors may be responsible for the development of HCC. Thus, it is necessary to perform further insight into the genes involved in hepatocarcinogenesis and to identify novel markers for HCC diagnosis and prognosis.

Aquaporins (AQPs) are a family of small (~30 kDa/monomer), hydrophobic, channel-forming membrane proteins that are expressed widely in the animal and plant kingdoms, and they are involved in the transepithelial fluid transport occurring in the urinary concentrating mechanism and glandular fluid secretion [4]. There are 13 members (AQP0~AQP12) having been identified so far in mammals. Among them, AQP0, AQP1, AQP2, AQP4, AQP5, AQP6, and AQP8 are primarily water selective, whereas AQP3, AQP7, AQP9, AQP10, and AQP12 also transport glycerol and possibly other small solutes. In addition to the classical function as osmotically driven transepithelial and transcellular water transporters, AQPs are also involved in swelling of tissues under stress, as in the injured cornea and the brain in stroke, tumor, and infection [5, 6]. In particular, accumulating evidence suggests the diagnostic and prognostic value of the aberrant expression of AQPs. AQP expression in some reports is associated with tumor progression. In liver tumors, Mazal et al. [7] demonstrated that AQP1 expression in HCC tissues was lower than that in cholangiocarcinoma, suggesting that AQP1 might be a highly selective marker for differentiated cholangiocytes and can be very helpful in the differential diagnosis of liver tumors; Padma et al. [8] reported that human HCC may be characterized by altered AQP9 expression and that AQP9 localization in the nontumourigenic liver mass may be dependent on underlying liver pathology; Jablonski et al. [9] found that AQP8 expression and AQP9 expression were significantly decreased in HCC versus normal liver. These findings implicated that the AQPs might play an important role in human HCC.

Of 13 AQP varieties, AQP1, -8, and -9 were downregulated in human HCC tissues [7–9]. The functional role of other AQP members in HCC has not been fully elucidated. Among them, AQP3, cloned in 1994, functions as a membrane channel of water and other small solutes such as glycerol and urea and plays a major role in fluid homeostasis [10]; AQP5, cloned from the salivary gland, is a 27-kDa protein that is known as an exocrinetype water channel with a unique tissue expression [11]. AQP3, that transports not only water but also glycerol and urea, is known to be expressed in kidney, skin, lung, and gastrointestinal tracts [10], and AQP5, that transports only water, is known to be expressed in various organs such as lung and saliva gland [12]. Recent studies have showed the altered expression of AQP3 and AQP5 in various tumor types. However, little is known about expression and precise role of the two proteins on HCC. Therefore, the aim of this study was to investigate the expression patterns and clinical significance of AQP3 and AQP5 in human HCC.

## 2. Materials and Methods 

### 2.1. Patients and Tissue Samples

The study was approved by the Research Ethics Committee of 302nd Hospital of PLA, Beijing, China. Informed consent was obtained from all of the patients. All specimens were handled and made anonymous according to the ethical and legal standards.

A total of 130 patients with primary HCC who underwent a curative liver resection at the 302nd Hospital of PLA, Beijing, China, were included in this retrospective study. Tissues used in the study were retrieved from the tissue bank of the Department of Pathology at the 302nd Hospital of PLA. These patients were diagnosed as HCC between 2001 and 2006. None of the patients recruited in this study had chemotherapy or radiotherapy before the surgery. HCC diagnosis was based on the World Health Organization (WHO) criteria. Tumor differentiation was defined according to the Edmondson grading system. Liver function was assessed using the Child-Pugh scoring system. Tumor staging was determined according to the sixth edition of the tumor-node-metastasis (TNM) classification of the International Union against Cancer. The clinicopathological features of 130 patients are summarized in Table 1. 

The median follow-up period was 8.6 years. Postoperative surveillance included routine clinical and laboratory examinations every third month, computed tomography scans of the abdomen, and radiographs of the chest every third month. After 5 years, the examination interval was extended to 12 months.

### 2.2. Immunohistochemistry Analysis

AQP3 expression and AQP5 expression were immunohistochemically evaluated in paraffin-embedded specimens of 130 patients with HCC. Surgical specimens were fixed in 10% formalin, embedded in paraffin, and sectioned at a 4 *μ*m thickness. For heat-induced epitope retrieval, deparaffinized sections were soaked in 10 mM citrate buffer (pH 6.0) and treated at 95°C for 30 min using the microwave oven method. Immunohistochemical staining was performed using the avidin-biotin immunoperoxidase technique according to our previous studies [13–15]. The activity of endogenous peroxidase was blocked by incubation with 0.3% H_2_O_2_ in methanol for 15 min, and nonspecific immunoglobulin binding was blocked by incubation with 10% normal goat serum for 10 min. Sections were incubated at room temperature for 4 h with anti-AQP3 rabbit polyclonal antibody (#sc-20811, Santa Cruz Biotechnology, Inc., USA) or with anti-AQP5 rabbit polyclonal antibody (#sc-28628, Santa Cruz Biotechnology, Inc., USA) at a 1 : 100 or 1 : 150 dilution, and they were then rinsed and incubated for 30 min with a biotinylated second antibody. After washing, the sections were incubated for 30 min with horseradish peroxidase-conjugated streptavidin and were finally treated with 3,3′-diaminobenzidine tetrahydrochloride in 0.01% H_2_O_2_ for 10 min. The slides were counterstained with Meyer's hematoxylin. The negative controls were processed in a similar manner with PBS instead of primary antibody. The positive AQP3 expression and AQP5 expression confirmed by western blotting were used as positive controls for immunostaining.

Following a hematoxylin counterstaining, immunostaining was scored by two independent experienced pathologists, who were blinded to the clinicopathological parameters and clinical outcomes of the patients. The scores of the two pathologists were compared, and any discrepant scores were trained through reexamining the staining by both pathologists to achieve a consensus score. The number of positive-staining cells showing immunoreactivity in the cytoplasm and cell membrane for both AQP3 and AQP5 in ten representative microscopic fields was counted, and the percentage of positive cells was calculated. The percentage scoring of immunoreactive tumor cells was as follows: 0 (0%), 1 (1–10%), 2 (11–50%), and 3 (>50%). The staining intensity was visually scored and stratified as follows: 0 (negative), 1 (weak), 2 (moderate), and 3 (strong). A final score was obtained for each case by multiplying the percentage and the intensity score. Therefore, tumors with a multiplied score exceeding median of total scores for AQP3 or AQP5 were deemed to be low expressions of AQP3 or AQP5; all other scores were considered to be high expressions of AQP3 or AQP5.

### 2.3. Statistical Analysis

The software of SPSS version 13.0 for Windows (SPSS Inc., IL, USA) and SAS 9.1 (SAS Institute, Cary, NC) was used for statistical analysis. The chi-squared test was used to show differences in categorical variables. Correlations between AQP3 expression and AQP5 expression were calculated using Spearman's correlation. Patient survival and the differences in patient survival were determined by the Kaplan-Meier method and the log-rank test, respectively. A Cox regression analysis (proportional hazard model) was performed for the multivariate analyses of prognostic factors. Differences were considered statistically significant when *P* was less than 0.05.

## 3. Results

### 3.1. Expression Patterns and Subcellular Localization of AQP3 and AQP5 Proteins in HCC

**Figure 1:**
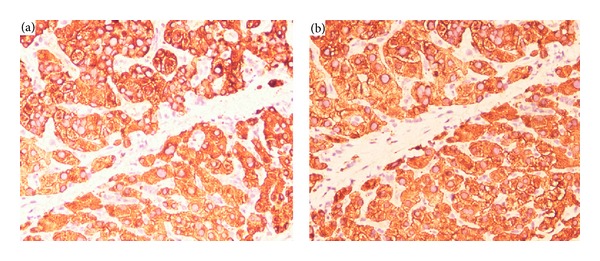
Representative immunohistochemical images of AQP3 (a) expression and AQP5 (b) expression in HCC tissues (original magnification ×400). AQP3 and AQP5 positive staining results were both indicated by numerous yellowish granules in the cytoplasm and membrane of tumor cells in HCC tissues.

The subcellular localization and the expression pattern of AQP3 and AQP5 proteins in 130 self-pairs of HCC and adjacent nonneoplastic liver tissues were observed by the immunohistochemistry analysis. As shown in Figure 1, both AQP3 positive staining and AQP5 positive staining were localized in the cytoplasm and membrane of tumor cells in HCC tissues. Compared with the adjacent nonneoplastic tissues, the immunohistochemistry scores of AQP3 (mean ± S.D.: 5.61 ± 0.23 versus 2.18 ± 0.09, *P* < 0.001) and AQP5 (mean ± S.D.: 6.28 ± 0.36 versus 2.16 ± 0.05, *P* < 0.001) proteins were both significantly increased in HCC tissues. In addition, the expression levels of AQP3 and AQP5 in 130 HCC cases were summarized in Table 2. Based on the scoring system used in the present study, 52 (40.00%) cases were both high expression of AQP3 and AQP5, 27 (20.77%) cases were both low expression of AQP3 and AQP5, 30 (23.08%) cases were AQP3 high and AQP5 low expression, and 21 (16.15%) cases were AQP3 low and AQP5 high expression. As determined by Spearman's correlation, the AQP3 expression was significantly associated with the AQP5 expression (*r* = 0.76, *P* = 0.01, Table 2).

### 3.2. Association of AQP3 and AQP5 Protein Expression with the Clinicopathological Features of HCC

To evaluate whether AQP3 protein expression and AQP5 protein expression were associated with clinicopathological features of patients with HCC, we correlated immunohistochemical AQP3 and AQP5 staining results with tumor stage, tumor grade, serum AFP level, presence of cirrhosis, and underlying liver disease including alcohol abuse, viral hepatitis B and C, sex, and age (Table 1). According to the results, we found that the expression levels of AQP3 protein in HCC tissues with the higher tumor stage (T3~4) and the positive serum AFP level were significantly lower than those with the lower tumor stage (T1~2, *P* = 0.005, Table 1) and the negative serum AFP level (*P* = 0.002, Table 1), respectively. In addition, the frequencies of aberrant AQP5 expression were higher in HCC tissues with higher tumor stage (T3~4) than those with lower tumor stage (*P* = 0.008, Table 1). AQP5 overexpression was also observed more frequently in HCC tissues with high tumor grade than those with low grade (*P* = 0.009, Table 1). Moreover, combined AQP3 and AQP5 protein expression was significantly associated with serum AFP (*P* = 0.008, Table 1), tumor stage (*P* = 0.006, Table 1), and tumor grade (*P* = 0.006, Table 1).

### 3.3. Prognostic Values of AQP3 and AQP5 Protein Expression in HCC

**Figure 2:**
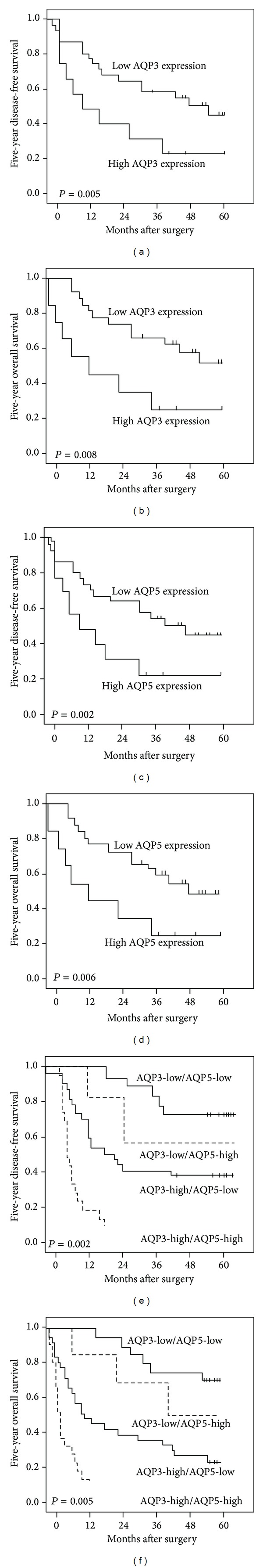
Disease-free survival and overall survival curves for two groups defined by low and high expression of AQP3 ((a) and (b)) or AQP5 ((c) and (d)) and for four groups defined by combined expression of AQP3 and AQP5 ((e) and (f)), in patients with HCC. The patients with high AQP3 and AQP5 expression had a significantly shorter 5-year overall and disease-free survival rate than those with low AQP3 and AQP5 expression (*P* = 0.006 and *P* = 0.01, resp.). In addition, the results by pairwise comparisons showed that the statistically significant difference of overall and disease-free survival existed between AQP3-high/AQP5-high patients and any of other three groups (*P* = 0.002 and 0.005, resp.). In all four groups, AQP3-high/AQP5-high patients had the poorest prognosis.

Five-year disease-free survival was observed in 30 (23.08%) patients, whereas in 100 (76.92%) patients, disease recurred, and 88 (67.69%) even died during a 5-year follow-up period. We observed a trend that 5-year disease-free survival in the group with high AQP3 expression was significantly poorer than that in the group with low AQP3 expression (*P* = 0.005, log-rank test; Figure 2(a)). Additionally, the Kaplan-Meier plot of 5-year overall survival curves stratified by AQP3 expression was shown in Figure 2(b). A significant relationship was found between AQP3 expression and 5-year overall survival (*P* = 0.008, log-rank test, Figure 2(b)). Similar with AQP3, the disease-free survival (Figure 2(c), *P* = 0.002) and overall survival (Figure 2(d), *P* = 0.006) of HCC patients with high AQP5 expression were both significantly shorter than those with low AQP5 expression. Moreover, the association between coexpression of AQP3/AQP5 and the survival rates was tested by the method of Kaplan-Meier. The Chi-square value by log-rank test (Mantel-Cox) indicated a significant difference among different groups with regard to the conjoined expression status of AQP3/AQP5 (Figures 2(e) and 2(f)). The results by pairwise comparisons showed that the statistically significant difference of disease-free survival and overall survival existed between AQP3-high/AQP5-high patients and any of other three groups (*P* = 0.002 and 0.005, resp.). In all four groups, AQP3-high/AQP5-high patients had the poorest prognosis.

Furthermore, in a multivariate Cox model, including serum AFP, tumor stage, tumor grading, presence of cirrhosis, gender, age, AQP3 expression, AQP5 expression, and combined AQP3/AQP5 expression, we found that AQP3 expression (both *P* = 0.01, Table 3), AQP5 expression (*P* = 0.006 and 0.01, Table 3), and combined AQP3/AQP5 expression (*P* = 0.009 and 0.01, Table 3) were independent poor prognostic factors for both 5-year disease-free survival and 5-year overall survival in HCC.

## 4. Discussion

In the current study, we determined the expression patterns of AQP3 and AQP5 proteins in 130 HCC tissues and paired adjacent nonneoplastic tissues using immunohistochemistry analysis. We confirmed that the overexpression of AQP3 mainly occurred in the cytoplasm and cell membrane in HCC tissues relative to adjacent nonneoplastic tissues and that AQP5 expression was markedly upregulated in HCC tissues compared with paired adjacent nonneoplastic tissues. The increased expression of both AQP3 and AQP5 proteins was significantly associated with aggressive clinicopathological features of HCC. We observed the coexpression of AQP3 and AQP5 to be associated with tumor stage, tumor grade, tumor metastasis, and patient prognosis. Taken together, our results suggest for the first time that the coexpression of AQP3 and AQP5 proteins may be a useful diagnostic and prognostic marker in HCC patients. 

AQPs are water channel proteins that facilitate trans-cellular water movements [16]. Accumulating evidence suggests that AQPs are involved in cell migration and proliferation, adding them to an expanding list of effectors in tumor pathology. The aberrant expression of human AQPs has been reported to be associated with various cancers. In particular, Ishimoto et al. [17] demonstrated that the overexpression of both AQP3 and AQP5 was immunohistochemically observed on tumor cells in squamous cell carcinoma, whereas adenoid cystic carcinoma cells were faintly stained with those antibodies against AQPs; Guo et al. [13] indicated that the upregulation of AQP3 and AQP5 in lung cancer cells may be mostly associated with cellular differentiation; Based on RT-PCR analysis, Guo et al. [14] reported that AQP3 and AQP5 exhibited differential expression between human gastric carcinomas and corresponding normal tissues, which was confirmed by Western blot analyses. Guo et al. [15] found that the expression of AQP5 was significantly decreased ovarian cancer; High expression level of AQP3 was also observed by Kusayama et al. [18] in tumor areas of human primary squamous cell carcinoma such as esophageal and lingual cancers, and lymph node metastasis, but it was not observed in normal areas. In the present study, our immunohistochemistry analysis showed that AQP3 and AQP5 proteins were both upregulated in HCC tissues compared with the normal controls. These findings revealed the altered expression AQPs in several types of tumors upon their specific expression patterns.

In addition to the above expression studies, there have been some studies on the role of AQPs in human carcinogenesis that have been alluded. For example, Sekine et al. [19] found that the survival of biliary tract carcinoma patients with high AQP5 expression was longer compared to that of patients with low AQP5 expression. Cox's proportional hazard model revealed that AQP5 expression was an independent prognostic factor, and Chi-square analysis revealed that high AQP5 expression correlated to small tumor size in biliary tract carcinoma patients. AQP5 expression in colon cancer cell lines and human colon cancer tissues may be associated with cell proliferation and metastasis to liver [20]. Zhang et al. [21] showed that the high AQP5 protein expression in intestinal type of adenocarcinoma was significantly associated with lymph node metastasis and lymphovascular invasion in patients. Watanabe et al. [22] also found that upregulation of AQP5 might be involved in differentiation of human gastric cancer cells. Yang et al. [23] also have previously found that AQP5 expression in ovarian malignant and borderline tumors was significantly higher than that of benign ovarian tumors and normal ovarian tissue and that the increased AQP5 protein level was associated with lymph node metastasis and ascites. Li et al. [24] reported that AQP3 overexpression could facilitate colorectal carcinoma cell migration and that AQP3 may be considered a potential indicator and therapeutic target for colon tumor metastasis and prognosis. Otto et al. [25] indicated that loss of AQP3 protein expression in pT1 bladder cancer may play a key role in disease progression and is associated with worse progression-free survival. In this study, we also found that the overexpression of both AQP3 and AQP5 was associated with advanced tumor stage, positive distant metastasis, and unfavorable prognosis. Notably, patients with AQP3 overexpression in combination with AQP5 upregulation had a worse prognosis than all of the other patients. From these results, we suggest that AQP3 and AQP5 may serve as molecular prognostic markers for HCC and that AQP3 overexpression in combination with AQP5 upregulation may be associated with even worse prognosis of HCC patients. Overall, these results indicated that AQP3 and AQP5 are involved in the development of several tumor types, especially in HCC, but the two proteins function as tumor promoter or tumor suppressor in different tumor types.

In conclusion, our data suggest for the first time that the aberrant expression of AQP3 and AQP5 proteins may be strongly related to tumor progression and prognosis in patients with HCC. The overexpression of AQP3 in combination with upregulation of AQP5 may be an unfavorable prognostic factor for HCC. Although the role of AQP3 and AQP5 in human tumor pathology has been explored extensively, their molecular mechanisms in different tumor types have not been fully elucidated. Further studies are needed to investigate the precise mechanisms of AQP3 and AQP5 in the progression of HCC.

## Figures and Tables

**Table 1:** Association of AQP3 and AQP5 expression with clinicopathological features of 130 hepatocellular carcinoma patients.

Clinicopathological features	Case	AQP3-high (*n*, %)	*P *	AQP5-high (*n*, %)	*P *	AQP3-high/AQP5-high (*n*, %)	*P *
Age (years)							
≤50	72	47 (65.28)	NS	40 (55.56)	NS	30 (41.67)	NS
>50	58	35 (60.34)		33 (56.90)		22 (37.93)	
Gender							
Male	96	62 (64.58)	NS	54 (56.25)	NS	40 (41.67)	NS
Female	34	20 (58.82)		19 (55.88)		12 (35.29)	
Serum AFP							
Positive	72	64 (88.89)	0.002	43 (59.72)	NS	45 (62.50)	0.008
Negative	58	18 (31.03)		30 (51.72)		10 (17.24)	
Tumor stage							
T1	23	0 (0)	0.005	0 (0)	0.008	0 (0)	0.006
T2	40	21 (52.50)		17 (42.50)		10 (25.00)	
T3	52	46 (88.46)		40 (76.92)		27 (51.92)	
T4	15	15 (100.00)		15 (100.00)		15 (100.00)	
Tumor grade							
G1	31	20 (64.52)	NS	13 (41.94)	0.009	3 (9.68)	0.006
G2	76	49 (64.47)		40 (52.63)		29 (38.16)	
G3	23	13 (56.52)		20 (86.96)		20 (86.96)	
Growth pattern							
Trabecular	101	63 (62.38)	NS	54 (52.47)	NS	40 (39.60)	NS
Nontrabecular	29	19 (65.52)		19 (65.52)		12 (41.38)	
Cirrhosis							
Yes	86	46 (53.49)	NS	47 (54.65)	NS	34 (39.53)	NS
No	44	26 (59.09)		26 (59.09)		18 (40.91)	
Underlying liver disease							
Alcoholic	25	15 (60.00)	NS	15 (60.00)	NS	7 (28.0)	NS
Hepatitis B	49	28 (57.14)		28 (57.14)		18 (36.73)	
Hepatitis C	35	27 (77.14)		18 (51.43)		15 (42.86)	
Unknown	21	12 (57.14)		12 (57.14)		12 (57.14)	

Note: “NS” refers to that the differences among groups have no statistical significance.

**Table 2:** Expression of AQP3 and AQP5 proteins in 130 hepatocellular carcinoma patients.

	AQP5 expression		*P *
	High (*n* = 82)	Low (*n* = 48)	
AQP3 expression			
High (*n* = 73)	52	21	0.01
Low (*n* = 57)	30	27	

**Table 3:** Multivariate survival analysis of five-year overall and disease-free survival in 130 patients with hepatocellular carcinoma.

Features	Five-year overall survival			Five-year disease-free survival		
	HR	95% CI	*P *	HR	95% CI	*P *
Age	1.132	0.316–3.516	0.192	1.536	0.322–3.736	0.125
Gender	1.191	0.345–3.857	0.136	1.559	0.357–3.831	0.131
Serum AFP	1.931	0.685–4.056	0.063	1.953	0.615–4.273	0.062
Tumor stage	2.879	1.366–5.196	0.009	2.686	1.386–6.009	0.01
Tumor grade	1.563	0.609–4.088	0.081	1.551	0.607–4.466	0.086
Presence of cirrhosis	1.919	0.738–4.102	0.063	1.921	0.793–4.219	0.062
AQP3 expression	5.398	1.312–11.338	0.01	5.200	1.343–11.186	0.01
AQP5 expression	8.476	1.993–17.286	0.006	5.936	1.312–12.588	0.01
AQP3/AQP5 expression	6.982	1.601–15.193	0.009	5.695	1.381–11.902	0.01
